# AI inclusivity and the centrality of Africa: a systematic review of representation, innovation, and governance in global artificial intelligence

**DOI:** 10.3389/frai.2026.1881299

**Published:** 2026-08-28

**Authors:** Ramadile Moletsane

**Affiliations:** Department of Computer Sciences, Vaal University of Technology, Vanderbijlpark, South Africa

**Keywords:** Africa, AI bias, artificial intelligence, digital innovation, inclusivity

## Abstract

Artificial Intelligence (AI) is reshaping global systems across education, healthcare, finance, and agriculture. Despite its transformative potential, concerns about inclusivity persist, particularly regarding the underrepresentation of African contexts in AI development. This study examines Africa’s role in achieving globally inclusive AI and explores the implications of its marginalization. A qualitative systematic review design was adopted, guided by PRISMA protocols, synthesizing peer-reviewed literature, policy documents, and grey sources published between 2018 and 2025. The analysis was structured around a conceptual framework linking data diversity, development processes, and AI outcomes. Findings reveal that limited African representation in datasets, governance institutions, and innovation ecosystems contributes to algorithmic bias, reduced contextual relevance, and reinforcement of global digital inequalities. Conversely, African contributions, including context-sensitive innovation, sector-specific AI innovation, and ethical frameworks grounded in communal philosophies such as Ubuntu, demonstrate the continent’s centrality to equitable AI systems. Structural barriers such as infrastructure gaps, funding constraints, and unequal global partnerships continue to limit participation. The study concludes that AI inclusivity is unattainable without meaningful African engagement in data production, model development, and governance structures. Policy recommendations emphasize capacity building, data sovereignty, equitable partnerships, and strengthened continental AI strategies. Integrating Africa as a core participant is not only a moral imperative but a technical necessity for globally representative and socially responsive AI systems.

## 1 Introduction

Artificial Intelligence (AI) has emerged as one of the most transformative technologies of the 21st-century, reshaping economic systems, governance structures, education, healthcare, agriculture, and industrial production worldwide (Ahmad et al., 2025; Brandao, 2025). Recent advances in machine learning, generative AI, natural language processing, and predictive analytics have significantly accelerated the integration of AI into both public and private sectors (Brandao, 2025). Consequently, this rapid adoption has created new opportunities for innovation, productivity, and socio-economic development, as evidenced by large-scale economic projections and productivity gains associated with AI-enabled systems (Yuan et al., 2024). At the same time, as AI systems increasingly influence decision-making processes across societies, concerns regarding fairness, accountability, transparency, and inclusivity have become central to academic debates (Jobin et al., 2019). In response, these issues have also emerged as key priorities in policy and industry discussions, reflected in frameworks such as the OECD AI Principles and the NIST AI Risk Management Framework (NIST, 2023; OECD, 2019). The concept of AI inclusivity has gained prominence amid growing evidence that AI systems often reflect and reproduce existing social, economic, and political inequalities. Research on algorithmic bias has demonstrated that AI systems trained on incomplete or unrepresentative datasets may produce discriminatory outcomes across dimensions such as race, ethnicity, language, gender, and geography (Barocas and Selbst, 2016; Buolamwini and Gebru, 2018; Ferrara, 2024).

Similarly, emerging scholarship on digital colonialism and data governance argues that the development of AI remains heavily concentrated within a small number of countries, corporations, and leading research institutions, particularly those located in the Global North (Birhane, 2022; Couldry and Mejias, 2019). This concentration creates asymmetries in access to data, computational infrastructure, investment, and technical expertise, resulting in uneven participation in the production of AI knowledge, technologies, standards, and regulatory frameworks across the global landscape (Crawford, 2021; UNCTAD, 2021). Consequently, questions of who contributes data, who develops AI systems, who benefits from AI innovations, and who participates in governance processes have become increasingly important in discussions surrounding responsible and inclusive AI (Jobin et al., 2019; UNESCO, 2021). Within these debates, Africa occupies a particularly significant yet often underexamined position. Home to more than 1.4 billion people, thousands of languages, diverse cultural traditions, and rapidly expanding digital ecosystems, the continent represents one of the most important frontiers for future AI development (Bird, 2020; GSMA, 2024; United Nations, 2024). At the same time, numerous studies have highlighted persistent disparities in AI research capacity, computational infrastructure, funding availability, data representation, and participation in global governance structures across many African countries. These disparities raise concerns regarding the extent to which contemporary AI systems adequately reflect African realities, needs, and perspectives.

The implications of limited African participation extend beyond questions of regional representation. AI systems trained predominantly on data generated in the Global North may perform poorly when applied in African contexts, particularly in healthcare diagnostics, natural language processing, financial services, agriculture, and public administration. Furthermore, the underrepresentation of African languages, cultures, and socio-economic conditions in AI development processes may constrain the global applicability and fairness of emerging technologies (Setia and Monga, 2024). Scholars have therefore argued that meaningful inclusion requires not only broader demographic representation in datasets but also greater participation in innovation ecosystems, policy development, governance institutions, and knowledge production processes. At the same time, Africa is increasingly contributing to global AI innovation through the emergence of research networks, innovation hubs, startup ecosystems, and policy initiatives. Countries such as South Africa, Kenya, Nigeria, Rwanda, and Egypt have developed distinctive AI ecosystems characterized by varying levels of technological maturity, investment, and regulatory development (Oxford Insights, 2023). However, substantial differences in AI readiness, infrastructure, policy capacity, and innovation ecosystems persist across African countries, highlighting the need to avoid treating the continent as a homogeneous technological landscape (Birhane, 2022; UNCTAD, 2021).

Initiatives such as Deep Learning Indaba, Data Science Africa, and Zindi have strengthened AI capacity, local expertise, and context-sensitive innovation across Africa, supporting applications in healthcare, agriculture, education, and financial inclusion (Birhane, 2022; UNESCO, 2021). These developments suggest that Africa should be viewed not only as a recipient of AI technologies but also as an important source of innovation, knowledge, and alternative approaches to AI governance. Despite growing scholarly attention to AI ethics, governance, bias, and digital transformation, the literature remains fragmented, with limited synthesis of Africa’s contribution to inclusive AI ecosystems. Previous studies have frequently examined issues of algorithmic fairness, data colonialism, governance, innovation capacity, and technological adoption as largely separate areas of inquiry (Birhane, 2022; Couldry and Mejias, 2020; Jobin et al., 2019).

Consequently, there remains a limited understanding of how African representation, innovation, and participation in governance collectively influence the development of globally inclusive AI systems (Smith and Neupane, 2018; UNESCO, 2021). Existing studies have tended to examine issues such as algorithmic fairness, data governance, innovation capacity, and digital transformation independently, resulting in fragmented knowledge regarding Africa’s role in inclusive AI development (Birhane, 2022; Jobin et al., 2019). Despite growing scholarly interest in AI ethics, governance, innovation, and algorithmic fairness, there is limited synthesis on how these dimensions interact to shape AI inclusivity in African contexts (Birhane, 2022; UNCTAD, 2021). As a result, policymakers, researchers, and practitioners lack a holistic understanding of the factors that facilitate or constrain meaningful African participation across the AI lifecycle. Addressing this knowledge gap is essential for informing the development of fair, representative, and globally relevant AI systems (OECD, 2019; UNESCO, 2021).

To address this gap, this study undertakes a systematic review of the literature on AI inclusivity in African contexts. The review seeks to synthesize existing evidence concerning representation, innovation, governance, and structural barriers affecting African participation in AI ecosystems. By integrating insights across these interconnected dimensions, the study contributes to ongoing debates regarding the conditions necessary for developing fair, representative, and globally relevant AI systems. Accordingly, the objective of this study is to systematically review and synthesise the existing literature on AI inclusivity in African contexts. Specifically, the review examines issues of representation and algorithmic fairness, innovation capacity and contextual adaptation, ethical and governance considerations, and structural barriers influencing African participation in AI ecosystems. The study is guided by the following research questions:

*RQ1:* How is Africa represented within contemporary AI datasets, development processes, and governance structures?

*RQ2:* What contributions do African innovation ecosystems make toward inclusive and contextually relevant AI development?

*RQ3:* What structural barriers constrain African participation in global AI ecosystems?

*RQ4:* What implications do these findings have for the development of fair, representative, and globally inclusive AI systems?

The remainder of the paper is organised as follows. The theoretical and conceptual framework(s) for the study are addressed in Section 2. Followed by the literature review in Section 3. The methodology is discussed in Section 4 and data analysis in the Section 5. The results and the discussion are presented in Section 6. This study concludes in Section 7.

## 2 Theoretical and conceptual framework

### 2.1 Theoretical framework

This study is theoretically informed by Decolonial Theory (Mignolo, 2011; Quijano and Ennis, 2000) and Socio-Technical Systems Theory (STS) (Bostrom and Heinen, 1977; Trist and Bamforth, 1951). Together, these perspectives provide a comprehensive framework for examining how power, representation, participation, and inclusivity shape contemporary AI ecosystems and Africa’s emerging role within them. Decolonial Theory highlights how historical and contemporary structures of power continue to influence knowledge production, technological development, and participation in global innovation systems (Mignolo, 2011; Quijano and Ennis, 2000). In contrast, STS emphasizes that technological outcomes emerge through interactions between technical infrastructures and the social, institutional, political, economic, and cultural contexts in which they are embedded (Bijker et al., 1987; Geels, 2004). The combined application of these theories enables a critical analysis of the structural and contextual factors that influence equitable participation in AI development, governance, and innovation.

Decolonial Theory provides a critical lens for understanding how historical structures of colonial power continue to influence contemporary knowledge production, technological development, and global governance. According to Quijano and Ennis (2000), coloniality persists beyond formal colonialism through enduring systems of power, knowledge, and social organization that privilege particular epistemologies while marginalizing others. Mignolo (2011) further argues that modern knowledge systems continue to be shaped by Eurocentric assumptions that often exclude alternative perspectives originating from the Global South. Within AI scholarship, decolonial perspectives have increasingly been applied to critiques of data colonialism, algorithmic bias, digital dependency, and unequal participation in technological innovation (Couldry and Mejias, 2019). From this perspective, the concentration of AI research, infrastructure, investment, and governance within a limited number of countries and institutions risks reproducing historical asymmetries in knowledge generation, resource allocation, and decision-making authority. Consequently, the underrepresentation of African languages, datasets, cultural contexts, and epistemologies within AI systems can be interpreted not merely as a technical limitation but as a manifestation of broader structural inequalities embedded within global technological systems.

Complementing this perspective, STS emphasizes that technological outcomes emerge through dynamic interactions between technical infrastructures and the social, institutional, cultural, economic, and political environments in which they operate. Originating from the work of Trist and Bamforth (1951), STS posits that technological and social systems are mutually constitutive and must be jointly optimized to achieve effective outcomes. The theory rejects technological determinism by arguing that technologies are shaped by human actors, institutions, governance arrangements, and contextual conditions. Subsequent scholarship has reinforced the importance of examining the interactions between technological innovation, institutional structures, and societal actors in shaping socio-technical change (Geels, 2004). Applied to AI, this perspective suggests that inclusivity cannot be achieved solely through technical improvements in algorithms or datasets. Rather, it depends on broader socio-technical arrangements, including governance structures, institutional capacity, stakeholder participation, innovation ecosystems, regulatory frameworks, and societal values.

Drawing on these complementary theoretical foundations, this study conceptualizes AI inclusivity as the extent to which diverse populations, knowledge systems, languages, cultures, institutions, and governance actors are represented, empowered, and able to participate meaningfully throughout the AI lifecycle. This conceptualization is consistent with emerging international AI governance and ethics frameworks, which emphasize inclusiveness, diversity, fairness, participation, and equitable access as fundamental principles for responsible AI development (OECD, 2019; UNESCO, 2021). Inclusivity is therefore understood as both a technical and socio-political phenomenon that requires equitable representation and participation across multiple dimensions.

Four interrelated dimensions of AI inclusivity guide the analysis. First, data representation refers to the inclusion of diverse demographic, linguistic, cultural, and socio-economic groups within AI datasets and training resources, thereby reducing the risk of bias and exclusion. Second, innovation participation: concerns the involvement of African researchers, developers, entrepreneurs, institutions, and communities in AI research, design, development, and deployment. Third, governance inclusion: relates to meaningful participation in AI policy formulation, regulatory processes, ethical frameworks, and international governance mechanisms that shape the future direction of AI. Finally, societal relevance refers to the extent to which AI systems address context-specific developmental challenges and societal needs while promoting fairness, accountability, transparency, and equitable outcomes.

The integration of Decolonial Theory and STS provides a robust analytical framework for examining how Africa contributes to more inclusive AI ecosystems. While Decolonial Theory highlights issues of power, representation, epistemic justice, and historical inequalities, STS explains how inclusive outcomes emerge through interactions among technological systems, institutions, governance structures, and social actors. Together, these theories enable a critical assessment of the opportunities and challenges associated with Africa’s participation in shaping equitable, contextually relevant, and globally representative AI futures.

### 2.2 Conceptual framework

**Figure 1:**
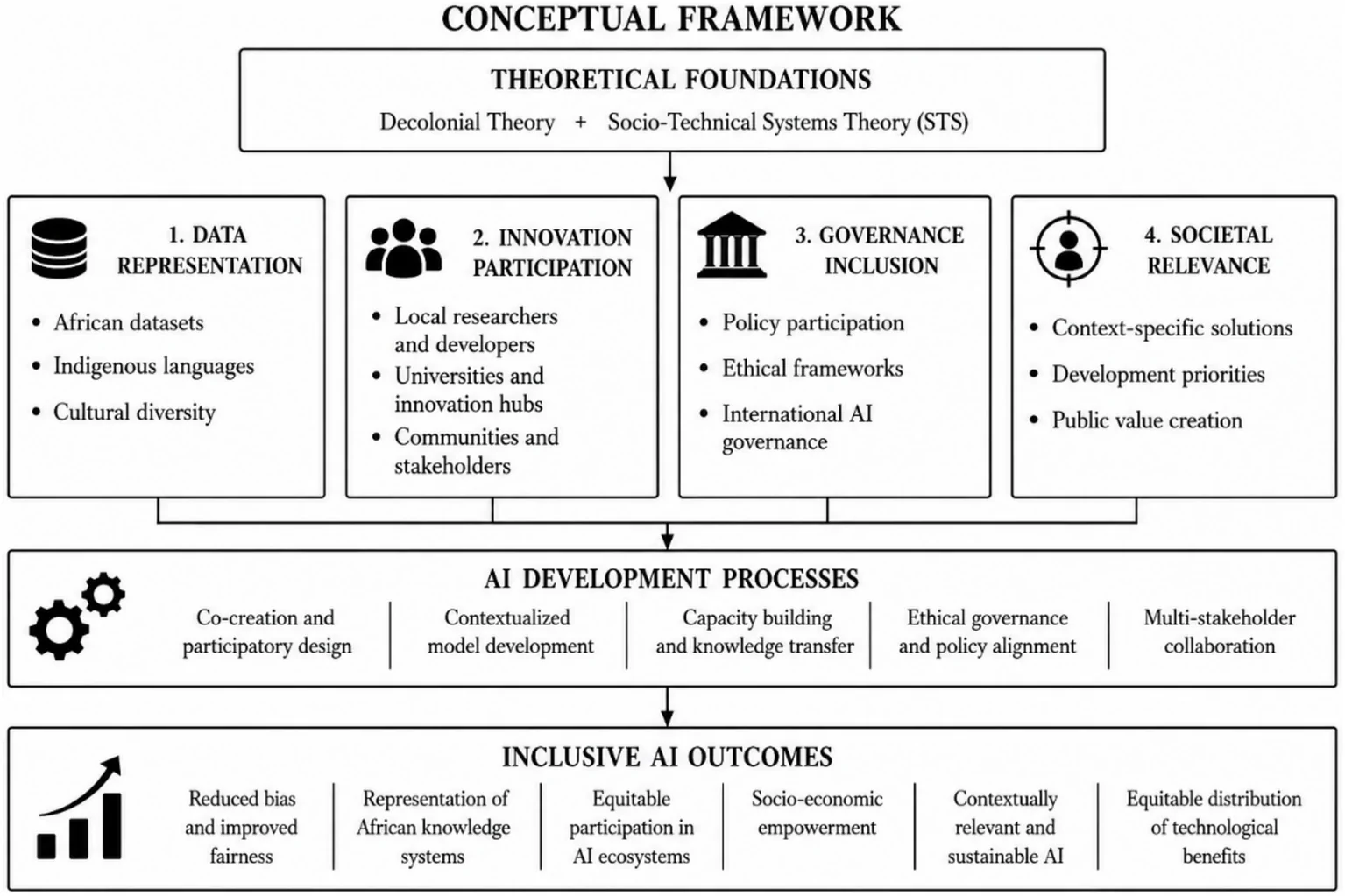
Conceptual framework for examining Africa’s role in promoting inclusive AI.

This study develops a conceptual framework for examining Africa’s role in promoting inclusive AI. The framework is derived from the principles of Decolonial Theory and STS, which collectively emphasize the importance of representation, participation, governance, and contextual relevance in technological development. As illustrated in Figure 1, the framework proposes that inclusive AI emerges through the interaction of four interrelated dimensions: data representation, innovation participation, governance inclusion, and societal relevance.

Data representation refers to the inclusion of diverse demographic, linguistic, cultural, and socio-economic groups within AI datasets and training resources (Buolamwini and Gebru, 2018). Representing African languages, local knowledge systems, and context-specific data is essential to reducing algorithmic bias and improving the relevance of AI applications (Pasipamire and Muroyiwa, 2024). Innovation participation concerns the active involvement of African researchers, developers, entrepreneurs, institutions, and communities in AI research, design, development, and deployment. Greater participation strengthens local ownership of technological innovation and contributes to more equitable knowledge production (Birhane, 2022; Smith and Neupane, 2018).

Governance inclusion focuses on the meaningful participation of African actors in AI policy development, regulatory processes, ethical frameworks, and international governance mechanisms. Inclusive governance ensures that diverse perspectives are incorporated into decisions that shape the development and use of AI technologies (OECD, 2019; UNESCO, 2021). Societal relevance refers to the extent to which AI systems address context-specific developmental challenges and societal priorities while promoting fairness, accountability, transparency, and equitable outcomes (Jobin et al., 2019; UNESCO, 2021).

The framework further proposes that these four dimensions collectively influence AI development processes, including co-creation and participatory design, contextualized AI development, capacity building, ethical governance, and multi-stakeholder collaboration. These processes serve as mechanisms for embedding inclusivity throughout the AI lifecycle. Ultimately, the framework suggests that inclusive AI development processes contribute to a range of inclusive AI outcomes, including reduced bias and improved fairness, greater representation of African knowledge systems, equitable participation in AI ecosystems, socio-economic empowerment, and the development of contextually relevant and sustainable AI solutions. Therefore, the framework provides an analytical lens for assessing how Africa contributes to the development of more inclusive, equitable, and globally representative AI ecosystems.

### 2.3 Literature review

### 2.4 AI inclusivity, representation, and data colonialism

The growing adoption of AI has heightened scholarly concern about inclusivity, fairness, and representation in AI systems. A central argument within contemporary AI scholarship is that the quality and diversity of training data significantly influence algorithmic performance and fairness outcomes. Studies consistently demonstrate that datasets used in AI development often underrepresent populations from the Global South, particularly African communities, languages, and socio-economic contexts (Quijano and Ennis, 2000). This imbalance has been associated with algorithmic bias, reduced model accuracy, and unequal technological outcomes across demographic groups.

Scholars examining data colonialism argue that the concentration of data collection, computational resources, and AI development within a limited number of countries and corporations reproduces historical patterns of global inequality. From this perspective, African communities frequently contribute data while remaining excluded from decision-making processes concerning data ownership, governance, and technological innovation. Birhane (2022) argues that the absence of African representation in AI datasets contributes to algorithmic exclusion, undermining fairness and contextual relevance. Similarly, research on facial recognition technologies shows that systems trained predominantly on non-African datasets often perform worse on darker-skinned populations.

Although the literature broadly agrees that representation matters for AI fairness, scholars differ regarding the extent to which technical interventions alone can resolve these challenges. While some studies emphasize dataset diversification, bias mitigation techniques, and algorithmic auditing as primary solutions to algorithmic discrimination (Buolamwini and Gebru, 2018; Ferrara, 2024), others argue that structural inequalities in knowledge production, data governance, and institutional power must also be addressed (Birhane, 2022; Couldry and Mejias, 2019). Consequently, inclusivity is increasingly understood not merely as a technical issue but as a socio-political challenge involving participation, power, and representation throughout the AI lifecycle (Jobin et al., 2019; UNESCO, 2021).

### 2.5 African AI innovation ecosystems

While discussions surrounding AI in Africa frequently focus on deficits and exclusion, an emerging body of literature highlights the continent’s growing role in technological innovation (Birhane, 2022; UNESCO, 2021). Across Africa, innovation hubs, research networks, startup ecosystems, and academic collaborations have contributed to the development of context-sensitive AI applications addressing local challenges in healthcare, agriculture, education, and financial services (Data Science Africa, 2024; Deep Learning Indaba, 2024; GSMA, 2024). However, African AI innovation is far from uniform. Significant variation exists across countries in terms of infrastructure, research capacity, policy development, and investment levels (Oxford Insights, 2023; UNCTAD, 2021). South Africa possesses one of the continent’s most developed AI research environments, supported by relatively strong higher education institutions, research infrastructure, and policy initiatives (Oxford Insights, 2023). Kenya has emerged as a leading center for digital innovation, particularly in agricultural technologies and mobile-based AI applications. Nigeria’s AI ecosystem has been driven largely by entrepreneurship, fintech innovation, and private-sector investment, contributing to the growth of AI-enabled digital services and startups (GSMA, 2024).

Rwanda has adopted a more state-led approach to AI development through national digital transformation programmes and strategic investments in emerging technologies, while Egypt has increasingly invested in national AI strategies, research capacity, and skills development (Oxford Insights, 2023; UNCTAD, 2021). These differences challenge representations of Africa as a homogeneous technological landscape. Instead, the literature suggests that multiple AI ecosystems are shaped by distinct political, economic, institutional, and regulatory conditions (Birhane, 2022; UNESCO, 2021). Despite variations in capacity and maturity, African innovation initiatives increasingly demonstrate that local actors are not merely consumers of imported technologies but active contributors to the development of contextually relevant AI solutions and alternative approaches to technological innovation (Birhane, 2022; Smith and Neupane, 2018).

### 2.6 AI governance, ethics, and Ubuntu

Questions of governance and ethics have become central to discussions of responsible AI. Existing governance frameworks have largely emerged from North American, European, and increasingly Asian policy environments, emphasizing principles such as transparency, accountability, privacy, explainability, and human rights (Jobin et al., 2019; OECD, 2019; UNESCO, 2021). While these principles remain important, scholars have questioned whether globally dominant frameworks adequately reflect the values, priorities, knowledge systems, and socio-economic realities of African societies (Birhane, 2022; Mhlambi, 2020; Mohamed et al., 2020). African AI scholars increasingly advocate for governance approaches that incorporate indigenous knowledge systems and locally grounded ethical perspectives (Birhane, 2022; Mohamed et al., 2020). Ubuntu philosophy has received particular attention as a framework for AI ethics because of its emphasis on relationality, communal well-being, mutual responsibility, and social interconnectedness (Metz, 2011; Mhlambi, 2020). Scholars argue that these principles offer valuable insights for addressing ethical challenges associated with AI deployment, particularly in contexts where collective welfare and community values are central to social organization.

The governance literature further highlights concerns regarding limited African participation in international AI regulatory processes. Although global initiatives have sought to establish common principles for the governance of AI, scholars argue that the development of AI governance frameworks has been dominated by actors from the Global North, resulting in the underrepresentation of African perspectives and priorities in international policy discussions (Floridi et al., 2018; Jobin et al., 2019).

Research further suggests that AI ethics and governance frameworks often reflect Western socio-cultural assumptions, raising concerns about their applicability to African contexts and reinforcing calls for more inclusive and participatory governance approaches (Mhlambi, 2020; Smith and Neupane, 2018). Consequently, scholars advocate for greater involvement of African policymakers, researchers, civil society organizations, and technology developers in global AI governance processes to ensure that regulatory frameworks address diverse cultural values, developmental priorities, and socio-economic realities. Researchers argue that meaningful participation from African stakeholders is essential for promoting context-sensitive governance approaches, addressing power imbalances in global AI development, and ensuring that AI systems reflect local needs and values rather than predominantly Western perspectives (Mhlambi, 2020; Mohamed et al., 2020). Similarly, Gwagwa et al. (2020) contend that inclusive AI governance requires active engagement from African governments, academia, industry, and civil society to foster responsible innovation and prevent the perpetuation of existing inequalities. Furthermore, (Okolo et al., 2023) emphasize that African participation in AI governance is necessary to address issues such as data governance, digital sovereignty, algorithmic fairness, and equitable access to the benefits of AI technologies.

### 2.7 Research gap

The literature reviewed demonstrates substantial progress in understanding AI representation, innovation, governance, ethics, and structural inequality in African contexts. However, existing studies have largely examined these dimensions independently. Research on algorithmic bias and fairness has primarily focused on data quality, representativeness, and technical mitigation strategies (Barocas and Selbst, 2016; Buolamwini and Gebru, 2018), while studies of innovation ecosystems have tended to emphasize technological development, entrepreneurship, and capacity building (Smith and Neupane, 2018; UNCTAD, 2021). Similarly, governance and ethics scholarship has focused on regulatory frameworks, ethical principles, and responsible AI without fully integrating analyses of innovation capacity, infrastructural inequalities, and participation in AI development (Jobin et al., 2019). Scholarship on AI in Africa has further highlighted issues of data colonialism, unequal participation, and structural exclusion, yet these concerns are often examined separately from broader questions of innovation and governance (Birhane, 2022; Mohamed et al., 2020). As a result, there remains limited systematic evidence regarding how representation, innovation, governance participation, and structural barriers collectively influence AI inclusivity in Africa. Addressing this gap requires an integrated analytical approach capable of synthesising insights across these interconnected dimensions. This study responds to that need by undertaking a systematic review of the relationships among representation, innovation, governance, and structural participation within African AI ecosystems and their implications for globally inclusive AI development.

## 3 Methodology

### 3.1 Research design

This study adopted a systematic qualitative literature review to synthesize existing evidence on Africa’s role in promoting inclusive AI. A systematic review was deemed appropriate because it provides a transparent, rigorous, and reproducible approach to identifying, evaluating, and synthesizing existing knowledge on a specific research problem, thereby facilitating the development of a comprehensive understanding of the subject and the identification of research gaps (Snyder, 2019; Tranfield et al., 2003). The review was conducted in accordance with the Preferred Reporting Items for Systematic Reviews and Meta-Analyses (PRISMA) guidelines developed by Page et al. (2021), which provides a structured, widely accepted framework for literature identification, screening, eligibility assessment, and study inclusion. The application of PRISMA enhances the transparency, consistency, and reliability of the review process while reducing the potential for selection bias and improving the reproducibility of findings. To address the research questions presented in the Introduction, this study adopted a systematic review methodology guided by the Preferred Reporting Items for PRISMA framework. The review followed a structured process comprising literature identification, screening, eligibility assessment, data extraction, quality appraisal, and thematic synthesis. This methodological approach ensured a transparent, rigorous, and reproducible synthesis of the literature on AI inclusivity and the centrality of Africa in global AI research.

### 3.2 Search strategy

A systematic search was conducted across multiple academic databases to identify relevant studies examining AI inclusivity, governance, innovation, ethics, representation, and participation within African contexts. The databases searched included: Scopus, Web of Science, IEEE Xplore, and Google Scholar. To ensure comprehensive coverage, keyword combinations and Boolean operators were used. The primary search string was: (“Artificial Intelligence” OR AI) AND (Africa OR African) AND (inclusivity OR inclusion OR fairness OR representation OR governance OR ethics). Additional search combinations included: (“AI governance” AND Africa), (“algorithmic bias” AND Africa), (“responsible AI” AND Africa), (“AI innovation” AND Africa), (“AI ethics” AND Africa), and (“data colonialism” AND Africa). Backward and forward citation tracking was also undertaken to identify additional relevant studies not captured through database searches.

### 3.3 Eligibility criteria

Studies were selected according to predefined inclusion and exclusion criteria.

#### 3.3.1 Inclusion criteria

Studies were included if they addressed AI, AI governance, AI ethics, algorithmic fairness, innovation ecosystems, or structural barriers to participation within African contexts. In addition, a limited number of foundational studies on AI fairness, bias, representation, and inclusivity were included, where they provided theoretical, conceptual, or analytical insights essential for understanding African participation in global AI systems. Although these studies were not exclusively conducted in African contexts, they were retained because they established influential concepts and frameworks that informed the interpretation of the African evidence. Eligible studies were published between 2018 and 2025, written in English, and available in full-text format through the selected databases.

#### 3.3.2 Exclusion criteria

Studies were excluded if they did not focus on AI-related issues, lacked relevance to African contexts or AI inclusivity, were published outside the specified period, or consisted of editorials, news articles, commentaries without substantive analytical content, or duplicate records.

### 3.4 Study selection process

**Figure 2:**
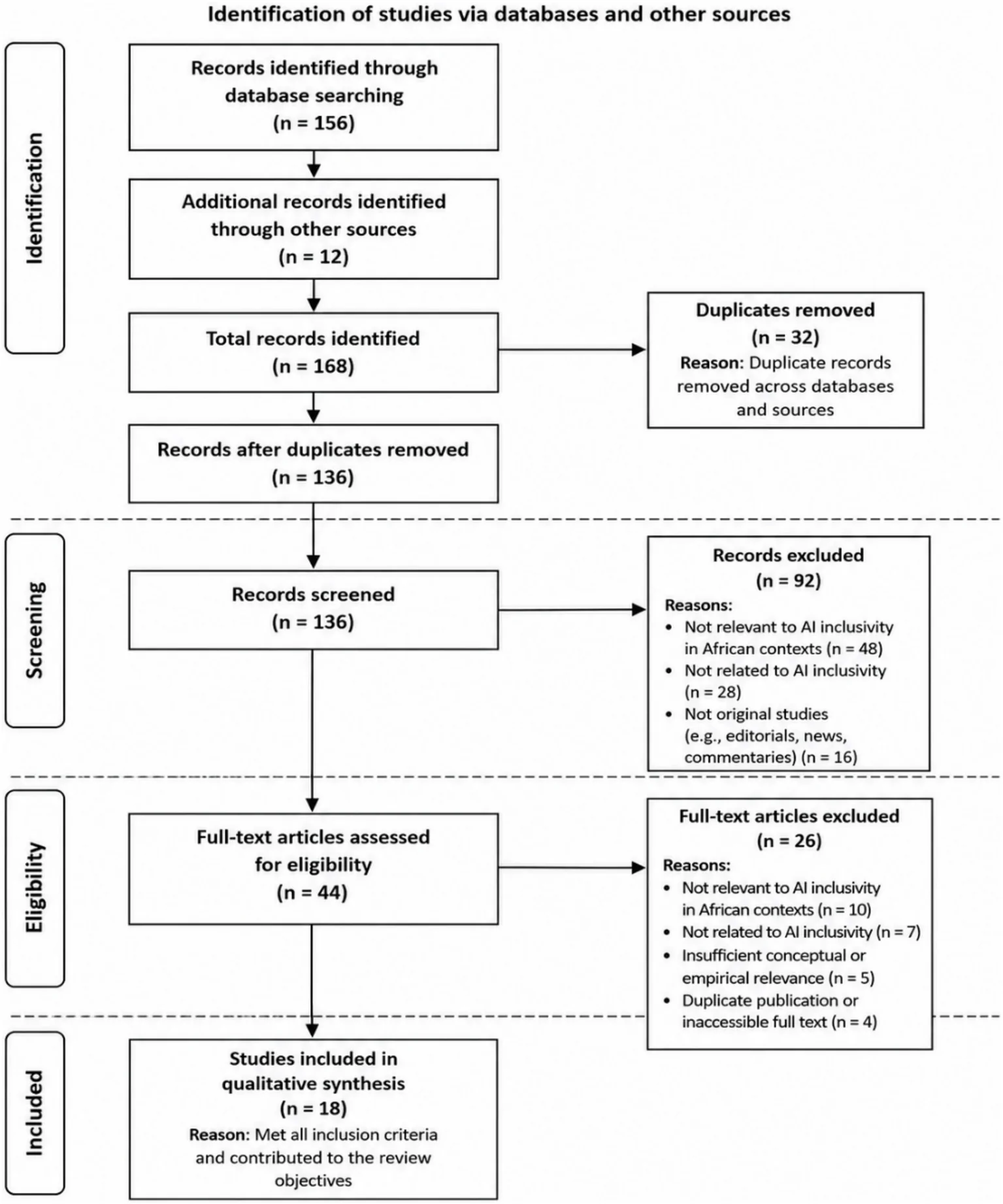
Prisma flow diagram.

The study selection process was conducted in accordance with the PRISMA guidelines to ensure transparency and methodological rigor. Following duplicate removal, title and abstract screening, and full-text eligibility assessment, 18 studies met the inclusion criteria and were retained for the final qualitative synthesis. Figure 2 presents the PRISMA flow diagram detailing the study selection process.

Following the full-text eligibility assessment, 26 studies were excluded because they did not satisfy the predefined inclusion criteria. The primary reasons for exclusion included a lack of relevance to AI inclusivity within African contexts, limited alignment with the review focus, insufficient conceptual or empirical relevance, and duplicate publications or inaccessible full texts. A summary of the exclusion reasons is presented in Table 1.

**Table 1:** Reasons for exclusion.

Reason for exclusion	*n*
Not relevant to AI inclusivity in African contexts	10
Not related to AI inclusivity	7
Insufficient conceptual or empirical relevance	5
Duplicate publication/inaccessible full text	4
Total	26

Table 2 summarises the study selection process undertaken in accordance with the PRISMA guidelines. The search strategy yielded 168 records from database searches and supplementary sources. Following duplicate removal, title and abstract screening, and full-text eligibility assessment, 18 studies met the inclusion criteria and were retained for the final qualitative synthesis.

**Table 2:** The PRISMA numbers table.

Stage of review process	Number of records
Records identified through database searching	156
Additional records identified through other sources	12
Total identified	168
Duplicates removed	32
Records screened	136
Records excluded at the screening stage	92
Full-text articles assessed for eligibility	44
Full-text articles excluded	26
Studies included in the final synthesis	18

### 3.5 Data extraction

Following the study selection process, data were manually extracted from the 18 included studies and entered into a structured Microsoft Excel spreadsheet using a predefined data extraction template. The extracted information included the author(s), publication year, study characteristics, methodological approach, geographical context, analytical focus, key findings, and relevance to the review objectives. Duplicate records identified across database searches were removed manually by comparing article titles, authors, publication years, and, where available, digital object identifiers (DOIs). The extracted data were subsequently used to facilitate thematic synthesis and comparative analysis. Table 3 presents a summary of the data extracted from the 18 studies included in the final qualitative synthesis.

**Table 3:** Characteristics of included studies.

No	Author(s)	Country/Regional focus	Study type	Thematic domain	Key contribution
1	Ferrara (2024)	Global (draws on literature and case from multiple countries, predominantly the United States)	Survey review	Representation and fairness	Synthesized sources and mitigation strategies for AI bias.
2	Grancia (2025)	Africa	Ethical analysis	Ethics and governance	Applied decolonial perspectives to AI ethics in African healthcare.
3	Birhane (2022)	Rwanda, Senegal, South Africa, Iceland, Finland, Sweden	Commentary	Representation and fairness	Highlighted exclusion of Black populations in AI datasets and ethics discourse.
4	Buolamwini and Gebru (2018)	Rwanda, Senegal, South Africa, Iceland, Finland, Sweden	Empirical study	Representation & fairness	Demonstrated racial and gender disparities in facial recognition systems.
5	Pasipamire and Muroyiwa (2024)	Africa	Review article	Representation & fairness	Examined algorithmic bias and AI trust within African contexts.
6	Yilma (2025)	Benin, Egypt, Ghana, Mauritius, Rwanda, and the African Union	Conceptual analysis	Ethics and governance	Examined Ubuntu and African AI governance initiatives.
7	Gwagwa et al. (2020)	Benin, Egypt, Ghana, Mauritius, Rwanda, and the African Union	Policy analysis	Governance and inclusion	Examined the benefits, challenges, and policy dimensions of AI deployment in Africa.
8	Ahmad et al. (2025)	Global	Conceptual study	Representation & Fairness	Integrated formal and socio- technical approaches to AI bias.
9	AL University (2025)	Sub-Saharan Africa	Research report	Innovation capacity	Assessed the AI ecosystem development across Sub-Saharan Africa.
10	Nsarkoh et al. (2025)	Africa	Technical framework	Innovation capacity	Proposed an Afrocentric AI training and renewal platform
11	Agamah et al. (2025)	Africa	Case study	Innovation capacity	Explored data science partnerships supporting African AI development.
12	Setia and Monga (2024)	India–Africa	Policy analysis	Governance and inclusion	Explored AI diplomacy and technological cooperation.
13	Kondo and Diwani (2023)	South Africa, Nigeria, Kenya, Ghana, Tanzania, Ethiopia, Uganda, Rwanda, Zimbabwe, and Senegal	Bibliometric analysis	Structural constraints	Mapped African AI research output and identified publication disparities
14	Beyene et al. (2024)	Angola, Benin, Botswana, Burundi, Burkina Faso, Côte d’Ivoire, Ethiopia, Gabon, Ghana, Kenya, Mauritania, Mauritius, Mozambique, Rwanda, Senegal, and Zimbabwe	Panel data analysis	Structural constraints	Investigated determinants of digital technology development.
15	Plantinga et al. (2024)	Benin, Botswana, Egypt, Kenya, Malawi, Mauritius, Mozambique, Namibia, Rwanda, South Africa, Zambia, and Zimbabwe	Policy study	Governance and Ethics	Examined responsible AI governance and policy learning across African countries.
16	Okolo et al. (2023)	Africa	Book chapter/ review	Governance and Responsible AI	Identified challenges and opportunities for responsible AI in Africa
17	Mensah and Van Wynsberghe (2025)	Africa	Conceptual analysis	Ethics and governance	Advocated the incorporation of Ubuntu values into African AI policy.
18	Aryee et al. (2025)	Ghana, Namibia, Rwanda, Kenya and Zambia	Cross-country survey	Structural constraints	Examined AI skills, infrastructure, governance, and workforce challenges.

The geographical distribution of the 18 included studies demonstrates that the evidence base is predominantly centred on Africa. Most of the included studies adopted a continental African perspective, while one study focused on Sub-Saharan Africa and another examined AI diplomacy between India and Africa. Several studies investigated multiple African countries, with South Africa, Rwanda, Ghana, Kenya, Zimbabwe, Senegal, Mauritius, Benin, Egypt, Namibia, and Zambia appearing most frequently across the included literature. Among the country-specific and multi-country studies, South Africa emerged as the most frequently represented country, followed by Rwanda, Ghana, Kenya, and Zimbabwe. In addition, three foundational studies adopted a global perspective to provide theoretical and empirical insights into algorithmic fairness, AI bias, and representation, thereby informing the interpretation of AI inclusivity within African contexts. Overall, the geographical distribution reflects a growing body of Africa-focused scholarship while demonstrating the value of selected global studies in contextualising AI inclusivity, governance, and innovation across the continent.

### 3.6 Quality assessment

To ensure methodological rigour, all included studies were evaluated based on: (i) Clarity of research objectives, (ii) Methodological transparency, (iii) Relevance to AI inclusivity and African contexts, (v) Quality of evidence presented, and (v) Contribution to understanding representation, governance, innovation, or inclusivity. Studies demonstrating substantial methodological limitations or insufficient relevance were excluded during the eligibility stage.

### 3.7 Data analysis

The final corpus of studies was analyzed using thematic synthesis. The analysis involved three stages. Stage 1: Open Coding: Included studies were read repeatedly and coded to identify recurring concepts, patterns, and relationships. Stage 2: Theme Development: Codes were grouped into broader thematic categories reflecting major areas of convergence and divergence within the literature. Stage 3: Interpretive Synthesis: Themes were interpreted through the study’s theoretical framework, informed by Decolonial Theory and Socio-Technical Systems Theory. Four dominant themes emerged: (i) Representation, Data Diversity, and Algorithmic Fairness. (ii) Innovation Capacity and Contextual Adaptation. (iii) Ethics, Governance, and Epistemic Inclusion. (iv) Structural Barriers and Digital Inequality. These themes provided the analytical foundation for examining how representation, participation, governance, and structural conditions collectively shape AI inclusivity within African contexts.

## 4 Data analysis

### 4.1 Representation, data diversity, and algorithmic fairness

Following the PRISMA-guided selection process, the final corpus of included studies was subjected to structured qualitative thematic synthesis. The purpose of the analysis was to systematically categorize the literature before interpreting the findings. A coding matrix was developed based on the study’s conceptual framework, which links input factors (representation and infrastructure), AI development processes (innovation and governance), and outcomes (fairness, inclusivity, and global applicability). Each included study was coded across the following analytical dimensions: thematic focus, methodological design, geographical focus, type of evidence provided, and key contribution to AI inclusivity discourse. The analysis revealed four dominant thematic clusters across the literature: (1) Representation and Data Diversity, (2) Innovation Capacity and Contextual Relevance, (3) Ethics and Governance Frameworks, and (4) Structural Barriers and Digital Inequality. Table 4 presents the thematic and methodological classification of included studies.

**Table 4:** Thematic and methodological classification of included studies.

Thematic domain	Key references	Methodological approach	Analytical focus	Contribution to the study’s conceptual framework
Representation and algorithmic fairness	Birhane (2022), Buolamwini and Gebru (2018), Ferrara (2024), Pasipamire and Muroyiwa (2024).	Empirical studies, Conceptual analyses, technical fairness assessments.	Dataset imbalance, racial bias, performance disparities, and fairness metrics	Demonstrates how limited African data inputs distort AI outcomes and undermine fairness
Innovation Capacity and Contextual Adaptation	Amankwah-Amoah and Adomako (2021), AL University (2025), Agamah et al. (2025), Nsarkoh et al. (2025)	Case studies; Policy analysis; Ecosystem reports.	Local AI ecosystems, resource-constrained innovation, Afrocentric AI models.	Establishes Africa as an active contributor to shaping context-sensitive AI development processes.
Ethics, Governance, and Epistemic Inclusion	Yilma (2025), Gwagwa et al. (2020), De Silva (2025), Setia and Monga (2024).	Normative ethics analysis, Policy review, Governance frameworks	Ubuntu ethics, regulatory participation, global AI diplomacy	Highlights the necessity of African philosophical and regulatory participation in shaping inclusive AI governance
Structural and Infrastructural Constraints	Okolo et al. (2023), Beyene et al. (2024), Kondo and Diwani (2023).	Empirical analysis, Bibliometric study, Developmental assessment	Funding disparities, infrastructure gaps, and publication imbalance	Identifies systemic barriers limiting Africa’s influence across AI input and development domains

Studies were classified based on their primary analytical contribution. Several studies intersected multiple domains; however, categorization reflects dominant thematic emphasis identified during coding.

The thematic analysis has identified four general patterns that cross through the literature under review. Firstly, there is systematic marginalization of data: the special inclusion of African data in standard AI systems is always accompanied by algorithmic bias and lower contextual accuracy. Second, new African centers of innovation also emerge in the literature, particularly in the agricultural industry, fintech, health, and education, as AI solutions have been demonstrated to be context-aware and resource-adaptive and are globally applicable. Third, the problem of governance asymmetry is not new, and Africa is underrepresented in international AI regulatory and standard-setting bodies, thereby reinforcing tendencies toward epistemic exclusion. Finally, structural factors (e.g., infrastructure deficits, funding disparities, and computational power) form a nexus that restricts Africa’s participation in AI development and regulation. Taken together, those trends support the study’s conceptual framework by showing that representation and participation directly affect AI inclusiveness.

## 5 Results and discussions

### 5.1 Representation, data diversity, and algorithmic fairness

This section addresses Research Question 1: *How is Africa represented within contemporary AI datasets, development processes, and governance structures?* The reviewed studies examine the extent to which African populations, languages, knowledge systems, and socio-cultural contexts are represented in AI research and development. The findings further explore how inadequate representation contributes to algorithmic bias, unequal AI outcomes, and the need for more inclusive datasets, governance mechanisms, and development practices.

The reviewed literature consistently identifies data representation as a critical determinant of AI inclusivity. Across the studies analysed, a recurring concern is the limited inclusion of African populations, languages, and socio-economic realities within datasets used to train contemporary AI systems (Birhane, 2022; Pasipamire and Muroyiwa, 2024). This underrepresentation has been associated with reduced algorithmic performance, biased outputs, and diminished contextual relevance when AI technologies are deployed in African settings (Buolamwini and Gebru, 2018; Ferrara, 2024).

A notable area of consensus concerns the relationship between dataset diversity and algorithmic fairness, with several studies arguing that more representative datasets contribute to improved model performance and equitable outcomes (Ahmad et al., 2025; Pasipamire and Muroyiwa, 2024). Studies examining facial recognition technologies, natural language processing systems, and predictive algorithms further demonstrate that models trained predominantly on Western datasets frequently exhibit reduced accuracy when applied to African populations (Birhane, 2022; Buolamwini and Gebru, 2018).

### 5.2 Innovation capacity and contextual relevance

This section addresses Research Question 2: *What contributions do African innovation ecosystems make toward inclusive and contextually relevant AI development?* The reviewed studies highlight Africa’s growing capacity to develop AI applications that respond to local socio-economic challenges across sectors such as agriculture, healthcare, education, and financial services. The discussion demonstrates how locally driven innovation contributes to contextually relevant AI while strengthening Africa’s role as an active contributor to global AI development. A second major theme concerns the growing contribution of African innovation ecosystems to AI development. Contrary to narratives portraying Africa primarily as a recipient of technological innovation, the reviewed studies provide evidence of increasing local capacity in AI research, entrepreneurship, and application development (Agamah et al., 2025; AL University, 2025; Nsarkoh et al., 2025). Furthermore, scholars highlight the development of Afrocentric AI frameworks and locally grounded innovation models that seek to align AI systems with African social, cultural, and developmental priorities (Nsarkoh et al., 2025).

These developments suggest that Africa is increasingly contributing not only as a site of AI deployment but also as a source of innovation, knowledge production, and technological adaptation (Setia and Monga, 2024). Across sectors such as agriculture, healthcare, education, and financial services, African innovators have developed context-sensitive AI solutions tailored to resource-constrained environments. These solutions include (i) AI-enabled precision agriculture and crop monitoring systems that support food security, (ii) clinical decision-support and disease surveillance applications for healthcare, (iii) adaptive learning platforms that enhance access to education, and (iv) AI-driven financial technologies that promote digital financial inclusion. Collectively, these innovations demonstrate Africa’s capacity to develop AI applications that respond to local socio-economic needs. They also contribute to the broader advancement of inclusive and contextually relevant AI.

Such innovations address challenges including limited internet connectivity, informal economic systems, rural service delivery, and linguistic diversity, demonstrating the ability of African researchers and developers to produce AI solutions that are both locally relevant and globally informative (Agamah et al., 2025; Gwagwa et al., 2020; Nsarkoh et al., 2025). Furthermore, scholars argue that locally developed AI systems are often better positioned to incorporate African cultural values, languages, and contextual realities into technological design and implementation (Grancia, 2025; Yilma, 2025). Importantly, the literature reveals significant variation across African countries. While countries such as South Africa, Kenya, Nigeria, Rwanda, and Egypt have developed relatively advanced AI ecosystems, other countries continue to face substantial challenges related to infrastructure, research capacity, investment, and policy readiness (AL University, 2025; Beyene et al., 2024; Kondo and Diwani, 2023; Setia and Monga, 2024). These findings suggest that Africa’s AI landscape is heterogeneous and characterised by diverse levels of technological development and innovation capacity. Recognizing this diversity is essential to understanding the opportunities and constraints of AI inclusivity across the continent.

### 5.3 Ethical frameworks, governance, and epistemic inclusion

This section addresses Research Questions 3 and 4: *What structural barriers constrain African participation in global AI ecosystems?* and *What implications do these findings have for the development of fair, representative, and globally inclusive AI systems?* The reviewed studies identify structural barriers—including unequal access to data, infrastructure, funding, governance, and research participation—that continue to limit Africa’s influence within global AI ecosystems. The discussion further examines the implications of these barriers for AI ethics, governance, epistemic inclusion, and the development of equitable, globally representative AI systems.

The literature identifies governance and ethics as critical dimensions of AI inclusivity. A recurring concern is that existing AI governance frameworks remain disproportionately influenced by institutions, regulatory traditions, and technological priorities located in the Global North (Birhane, 2022; Gwagwa et al., 2020; Plantinga et al., 2024). Several studies argue that this imbalance limits the representation of African perspectives in global AI decision-making processes and reduces the influence of African actors in shaping AI governance frameworks (Gwagwa et al., 2020; Okolo et al., 2023; Yilma, 2025). Decolonial scholars further argue that these asymmetries reproduce patterns of knowledge extraction and technological dependency, in which decisions on data governance, ethical standards, and AI development are often shaped outside African contexts (Birhane, 2022; Grancia, 2025). Consequently, AI inclusivity extends beyond technical fairness and requires meaningful participation of African actors in the governance structures that shape AI systems.

The literature further highlights the role of African ethical traditions in informing AI governance frameworks. Several studies identify Ubuntu as a valuable normative framework that emphasises relationality, collective wellbeing, human dignity, and communal responsibility, offering an alternative perspective to predominantly individualistic approaches commonly found in Western governance models (Mensah and Van Wynsberghe, 2025; Yilma, 2025). These scholars argue that incorporating African philosophies and local knowledge systems can contribute to more context-sensitive and culturally responsive governance arrangements, thereby strengthening epistemic inclusion and ensuring that AI systems better reflect the social realities of African communities.

Despite broad agreement regarding the need for greater African participation in AI governance, differences emerge concerning the most appropriate pathways toward inclusivity. Some studies advocate institutional reforms, policy coordination, governance capacity-building, and greater involvement in international standard-setting processes (Plantinga et al., 2024; Setia and Monga, 2024). Others call for more transformative decolonial approaches that challenge existing power structures underpinning AI development, data ownership, and knowledge production (Birhane, 2022; Grancia, 2025). Collectively, the literature suggests that achieving AI inclusivity requires not only technical safeguards and regulatory reforms but also governance frameworks that recognise diverse forms of knowledge, values, and participation in shaping the future of AI.

### 5.4 Structural barriers to African participation

This section addresses Research Question 3: *What structural barriers constrain African participation in global AI ecosystems?* The reviewed studies consistently identify several interconnected structural barriers that limit Africa’s meaningful participation in global AI ecosystems. These barriers include inadequate digital infrastructure, limited computational resources, insufficient investment in AI research and innovation, shortages of specialised AI expertise, and unequal access to research funding and international collaboration opportunities (Aryee et al., 2025; Beyene et al., 2024). Rather than functioning independently, these constraints reinforce one another, collectively reducing Africa’s capacity to develop, deploy, and govern AI technologies.

Limited digital infrastructure and computational resources hinder the development, training, and deployment of advanced AI models, while inadequate investment in research infrastructure constrains local innovation and reduces opportunities to develop contextually relevant AI solutions (AL University, 2025; Beyene et al., 2024). Skills shortages further weaken research capacity by limiting the availability of experienced AI researchers and practitioners capable of leading cutting-edge innovation. In addition, unequal access to funding and high-quality datasets limits the production of locally generated evidence and reduces Africa’s visibility in global AI research and development.

The reviewed studies also demonstrate that institutional and governance challenges extend beyond technical limitations. Fragmented governance frameworks, limited institutional capacity, and asymmetrical international collaborations frequently position African institutions as providers of data, contextual knowledge, or implementation support, while affording them comparatively little influence over research agendas, funding priorities, technology design, and global AI governance processes (Agamah et al., 2025; Gwagwa et al., 2020). This imbalance reinforces technological dependency and limits Africa’s ability to shape AI standards, policies, and innovation trajectories.

Despite these constraints, the reviewed studies identify promising pathways for strengthening African participation through sustained investment in digital infrastructure, AI education and skills development, regional research networks, equitable international partnerships, and contextually informed governance frameworks (Agamah et al., 2025; Nsarkoh et al., 2025; Plantinga et al., 2024). Taken together, the findings suggest that the principal challenge is not the presence of isolated barriers but the interaction of infrastructural, institutional, financial, and governance constraints that collectively shape Africa’s participation in global AI ecosystems. This integrated perspective is an important contribution of the present review. It demonstrates that meaningful AI inclusivity requires coordinated interventions across multiple dimensions rather than isolated technical solutions.

### 5.5 Why AI cannot be inclusive without Africa

Synthesising the findings across all themes reveals that AI inclusivity is shaped by the interaction of representation, innovation, governance, and structural participation (Aryee et al., 2025; Birhane, 2022; Gwagwa et al., 2020). The evidence suggests that meaningful African engagement contributes to improved dataset diversity, contextually relevant innovation, broader ethical perspectives, and more representative governance arrangements (Buolamwini and Gebru, 2018; Nsarkoh et al., 2025; Plantinga et al., 2024; Yilma, 2025). However, the review does not suggest that African participation alone guarantees inclusive AI outcomes. Rather, the findings indicate that continued underrepresentation may limit the fairness, legitimacy, and global applicability of AI systems, while inclusive outcomes also depend on governance quality, institutional capacity, and technical design choices (Ahmad et al., 2025; Ferrara, 2024; Okolo et al., 2023). Consequently, inclusivity emerges as a multidimensional process requiring participation throughout the AI lifecycle, from data generation and model development to governance and implementation. Viewed through the lenses of Decolonial Theory and Socio-Technical Systems Theory, the findings demonstrate that AI outcomes are shaped by both technical infrastructures and broader social relations, highlighting the need for greater equity in participation, knowledge production, and governance to achieve globally inclusive AI systems (Birhane, 2022; Grancia, 2025; Plantinga et al., 2024).

## 6 Conclusions and recommendations

### 6.1 Conclusion

This systematic review examined Africa’s role in promoting inclusive AI by analyzing literature on representation, innovation ecosystems, governance participation, and structural barriers. The findings indicate that Africa remains underrepresented in many dimensions of the global AI ecosystem, including data generation, research leadership, technological infrastructure, and participation in international governance processes.

At the same time, the review identifies growing evidence that African scholars, innovators, policymakers, and institutions are contributing to the development of contextually relevant AI. The reviewed studies highlight contributions through sector-specific innovations, regional AI governance initiatives, capacity-building programmes, collaborative research networks, and policy reforms. Together, these findings indicate an emerging shift from Africa being primarily a consumer of AI technologies to becoming an active contributor to inclusive, context-sensitive, and ethically informed AI development.

The review identified four interconnected themes shaping AI inclusivity: representation and data diversity; innovation capacity and contextual adaptation; ethics and governance; and structural barriers to participation. Collectively, these findings suggest that meaningful inclusion within AI systems extends beyond technical considerations and requires broader participation in knowledge production, policy formulation, technological development, and institutional decision-making. Importantly, the evidence indicates that limited African representation may constrain the fairness, contextual relevance, and global applicability of AI systems.

However, the findings also demonstrate considerable diversity across African countries in terms of innovation capacity, infrastructure development, governance maturity, and technological readiness. Consequently, Africa should not be understood as a homogeneous technological landscape but as a collection of diverse and evolving AI ecosystems. From the perspectives of Decolonial Theory and Socio-Technical Systems Theory, the findings highlight the importance of addressing both the technical and structural dimensions of inclusion. AI systems are shaped not only by algorithms and datasets but also by broader social, political, and institutional arrangements that determine whose knowledge, experiences, and priorities are reflected in technological development. Overall, the review suggests that strengthening African participation across the AI lifecycle may contribute to more representative, contextually relevant, and globally responsive AI systems. Achieving meaningful AI inclusivity requires sustained efforts to broaden participation, reduce structural inequalities, and promote diverse forms of knowledge and innovation.

### 6.2 Recommendations

Based on the findings of this review, several recommendations are proposed. (i) Strengthen AI Infrastructure and Research Capacity: Governments, development agencies, universities, and private-sector organisations should invest in high-performance computing infrastructure, research centres, innovation hubs, and data repositories to strengthen Africa’s AI development capacity. (ii) Promote Data Diversity and Data Sovereignty: Efforts should be made to increase the representation of African populations, languages, and socio-cultural contexts within AI datasets. Policies supporting data sovereignty, ethical data governance, and responsible data sharing should be prioritised to ensure equitable participation in AI development. (iii) Expand Participation in Global AI Governance: African governments, researchers, civil society organisations, and regional institutions should be supported to participate more actively in international AI governance initiatives. Greater representation may contribute to governance frameworks that better reflect diverse developmental and cultural contexts. (iv) Support Inclusive Innovation Ecosystems: Investment should be directed toward startups, research collaborations, and entrepreneurial initiatives that develop AI solutions tailored to local challenges. Partnerships should be based on principles of co-creation, shared ownership, and mutual benefit rather than extractive models of technological engagement. (v) Develop AI Education and Skills Programmes. Educational institutions should expand AI-related curricula, digital literacy programmes, and professional development initiatives to strengthen human capital and prepare future generations for participation in AI-driven economies.

### 6.3 Limitations and future research

This study has several limitations. First, the review was restricted to English-language publications and therefore may not capture relevant research published in other languages. Second, the analysis focused primarily on studies published between 2018 and 2025, potentially excluding earlier foundational work. Third, as a qualitative systematic review, the study synthesises existing evidence rather than generating new empirical data. Future research should undertake comparative analyses across African regions and countries to better understand variations in AI development, governance, and innovation capacity. Additional empirical studies are also needed to examine the long-term impacts of African participation in global AI ecosystems and to evaluate emerging governance models grounded in African ethical traditions and socio-cultural contexts.

## Data Availability

The original contributions presented in the study are included in the article/supplementary material, further inquiries can be directed to the corresponding author.
